# Supplementary material for: A Global Screen for Assembly State Changes of the Mitotic Proteome by SEC-SWATH-MS
Source: Cell Syst. 2020 Feb 26;10(2):133–155.e6. doi: 10.1016/j.cels.2020.01.001 (PMC7042714; doi:10.1016/j.cels.2020.01.001)

Q53LP3 | SWAHC_HUMAN | SOWAHC ANKRD57 C2orf26
Monomer MW [kDa]: 55.672 Monomer expected elution fraction: 45

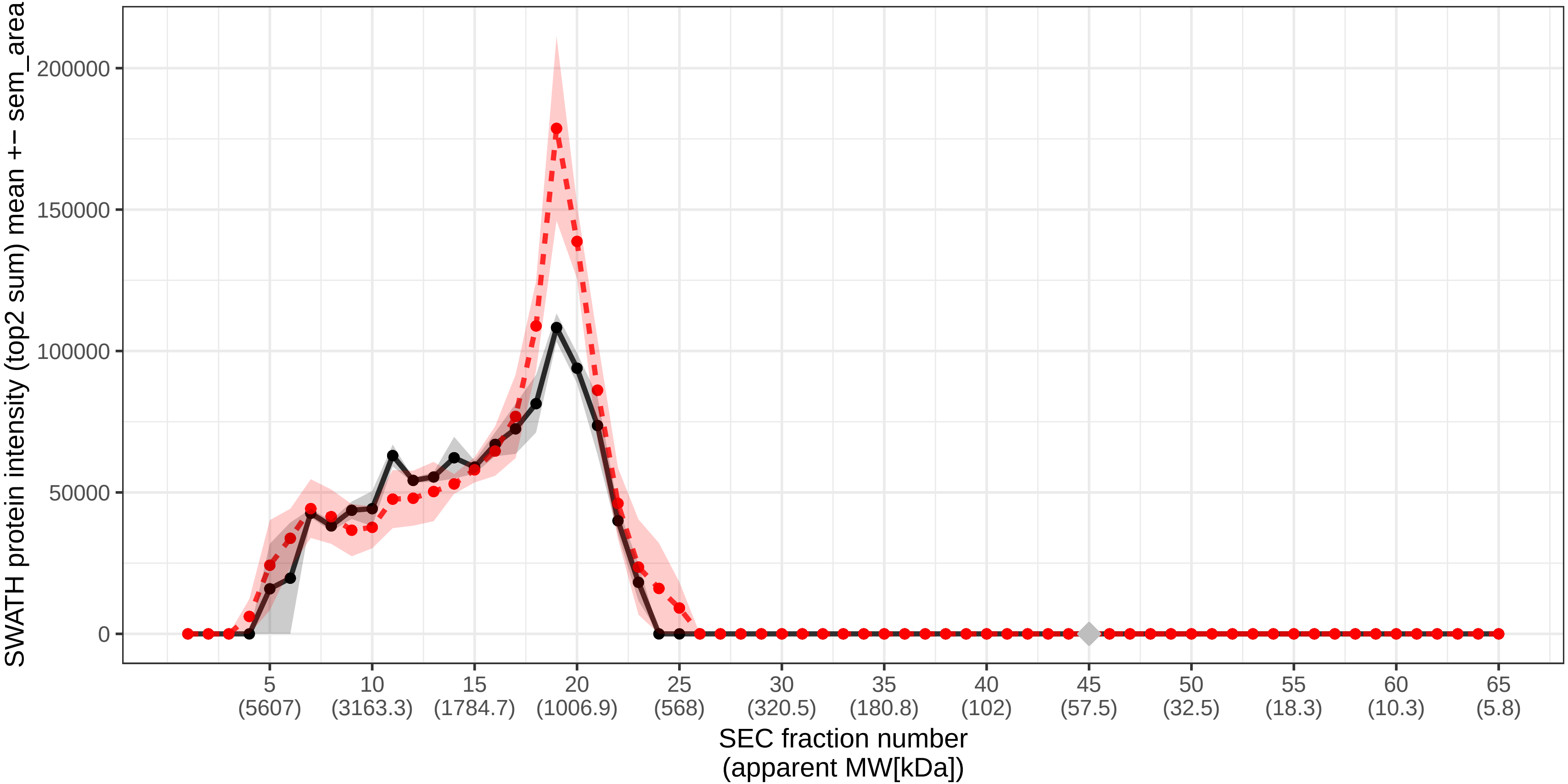

Supplement: Data S1. SEC-SWATH-MS Protein Chromatograms, Related to Figure 1 [file mmc6.zip › SECchrom_Q53LP3_SWAHC_HUMAN_SOWAHC_ANKRD57_C2orf26.pdf]
